# Introduction of primary screening using high-risk HPV DNA detection in the Dutch cervical cancer screening programme: a population-based cohort study

**DOI:** 10.1186/s12916-019-1460-0

**Published:** 2019-12-11

**Authors:** Clare A. Aitken, Heleen M. E. van Agt, Albert G. Siebers, Folkert J. van Kemenade, Hubert G. M. Niesters, Willem J. G. Melchers, Judith E. M. Vedder, Rob Schuurman, Adriaan J. C. van den Brule, Hans C. van der Linden, John W. J. Hinrichs, Anco Molijn, Klaas J. Hoogduin, Bettien M. van Hemel, Inge M. C. M. de Kok

**Affiliations:** 1 Department of Public Health, Erasmus MC University Medical Center, Dr. Molewaterplein 40, 3015 CN Rotterdam, the Netherlands; 2 PALGA, the nationwide network and registry of histo- and cytopathology in the Netherlands, De Bouw 123, 3991 SZ Houten, the Netherlands; 3 Department of Pathology, Radboud University Medical Center, P.O. Box 9101, 6500 HB Nijmegen, the Netherlands; 4 Department of Pathology, Erasmus MC University Medical Center, Dr. Molewaterplein 40, 3015 CN Rotterdam, the Netherlands; 5 Division of Clinical Virology, Department of Medical Microbiology, The University of Groningen, University Medical Center Groningen, Hanzeplein 1, 9713 GZ Groningen, the Netherlands; 6 Department of Medical Microbiology, Radboud University Medical Center, P.O. Box 9101, 6500 HB Nijmegen, the Netherlands; 7 Facilitaire Samenwerking Bevolkingsonderzoeken, Godebaldkwartier 435, 3511 DT Utrecht, the Netherlands; 8 Department of Medical Microbiology, University Medical Center Utrecht, Heidelberglaan 100, 3584 CX Utrecht, the Netherlands; 9 Jeroen Bosch Hospital, Pathologie-DNA, Henri Dunantstraat 1, 5223 GZ ‘s-Hertogenbosch, the Netherlands; 10 Symbiant Pathology Expert Centre Hoorn (Westfriesgasthuis), Maelsonstraat 3, 1624 NP Hoorn, the Netherlands; 11 Department of Pathology, University Medical Center Utrecht, Heidelberglaan 100, 3584 CX Utrecht, the Netherlands; 12 NMDL-LCPL, Visseringlaan 25, 2288 ER Rijswijk, the Netherlands; 13 Department of Pathology and Medical Biology, the University of Groningen, University Medical Center Groningen, Hanzeplein 1, 9713 GZ Groningen, the Netherlands

**Keywords:** Cervical cancer screening, hrHPV screening, Population-based screening, Cancer screening programmes

## Abstract

**Abstract:**

**Background:**

In January 2017, the Dutch cervical cancer screening programme transitioned from cytomorphological to primary high-risk HPV (hrHPV) DNA screening, including the introduction of self-sampling, for women aged between 30 and 60 years. The Netherlands was the first country to switch to hrHPV screening at the national level. We investigated the health impact of this transition by comparing performance indicators from the new hrHPV-based programme with the previous cytology-based programme.

**Methods:**

We obtained data from the Dutch nationwide network and registry of histo- and cytopathology (PALGA) for 454,573 women eligible for screening in 2017 who participated in the hrHPV-based programme between 1 January 2017 and 30 June 2018 (maximum follow-up of almost 21 months) and for 483,146 women eligible for screening in 2015 who participated in the cytology-based programme between 1 January 2015 and 31 March 2016 (maximum follow-up of 40 months). We compared indicators of participation (participation rate), referral (screen positivity; referral rate) and detection (cervical intraepithelial neoplasia (CIN) detection; number of referrals per detected CIN lesion).

**Results:**

Participation in the hrHPV-based programme was significantly lower than that in the cytology-based programme (61% vs 64%). Screen positivity and direct referral rates were significantly higher in the hrHPV-based programme (positivity rate: 5% vs 9%; referral rate: 1% vs 3%). CIN2+ detection increased from 11 to 14 per 1000 women screened. Overall, approximately 2.2 times more clinical irrelevant findings (i.e. ≤CIN1) were found in the hrHPV-based programme, compared with approximately 1·3 times more clinically relevant findings (i.e. CIN2+); this difference was mostly due to a national policy change recommending colposcopy, rather than observation, of hrHPV-positive, ASC-US/LSIL results in the hrHPV-based programme.

**Conclusions:**

This is the first time that comprehensive results of nationwide implementation of hrHPV-based screening have been reported using high-quality data with a long follow-up. We have shown that both benefits and potential harms are higher in one screening round of a well-implemented hrHPV-based screening programme than in an established cytology-based programme. Lower participation in the new hrHPV programme may be due to factors such as invitation policy changes and the phased roll-out of the new programme. Our findings add further to evidence from trials and modelling studies on the effectiveness of hrHPV-based screening.

## Background

Primary high-risk HPV (hrHPV) DNA screening, evaluated in clinical trials, has been shown to be more effective and cost-effective than cytology screening for the detection of pre-malignant and malignant cervical lesions [1, 2]. Following advice from the Dutch Health Council [3] and a feasibility study by the Dutch National Institute for Public Health and the Environment (RIVM) [4], primary hrHPV screening replaced cytology screening in the Dutch national cervical cancer screening programme in January 2017. Each of the five regional screening organisations implemented hrHPV-based screening sequentially during the first quarter of 2017, and by April 2017, the national implementation was complete. Women can choose either to have a cervical smear taken by their general practitioner (GP) or to use a self-sampling kit [5]. Laboratory testing of screening programme samples is performed in five dedicated screening laboratories.

As part of the initial feasibility study, modelling analysis was conducted assessing the costs and effects of implementing primary hrHPV-based screening in the Netherlands [4]. Recent modelling estimated that nationwide implementation of primary hrHPV-based screening was expected to reduce cervical cancer diagnoses by 13% and related deaths by 15% compared with cytology-based screening, while also reducing overall programme costs [6].

The success of a screening programme depends on the implementation of well-defined protocols and guidelines [7]. Screening programmes should be regularly monitored using high-quality data for quality assurance, to evaluate effectiveness and to identify potential harms [8]. Although results from the implementation of primary hrHPV screening in Italy and Turkey have been published [9, 10], these data lack robust results on detection of cervical intraepithelial neoplasia (CIN) lesions and do not compare the performance of hrHPV screening with cytology-based screening. Results from the Italian programme were also limited to a number of regions. Comprehensive results from the implementation of a nationwide hrHPV screening programme have yet to be published.

Data from the Dutch nationwide network and registry of histo- and cytopathology (PALGA) has enabled regular, high-quality monitoring of organised cervical cancer screening in the Netherlands for many years. This comprehensive dataset has national coverage [11], enabling us to assess the impact of cervical cancer screening programme policies on a national level. In order to evaluate the performance of the new primary hrHPV-based screening programme, we aimed to compare outcomes of the first year of the new programme with outcomes of the previous cytology-based cervical cancer screening programme.

## Methods

### The cytology-based Dutch cervical screening programme

Until the end of 2016, the Dutch cervical cancer screening programme used cytology as the primary screening test. Women were invited to make an appointment for screening with their GP every 5 years from ages 30 to 60. Women could choose to opt out of screening either temporarily (in the case of pregnancy, illness or other short-term reasons) or indefinitely (in the case of hysterectomy or non-medical reasons such as conscientious objection).

**Fig. 1:**
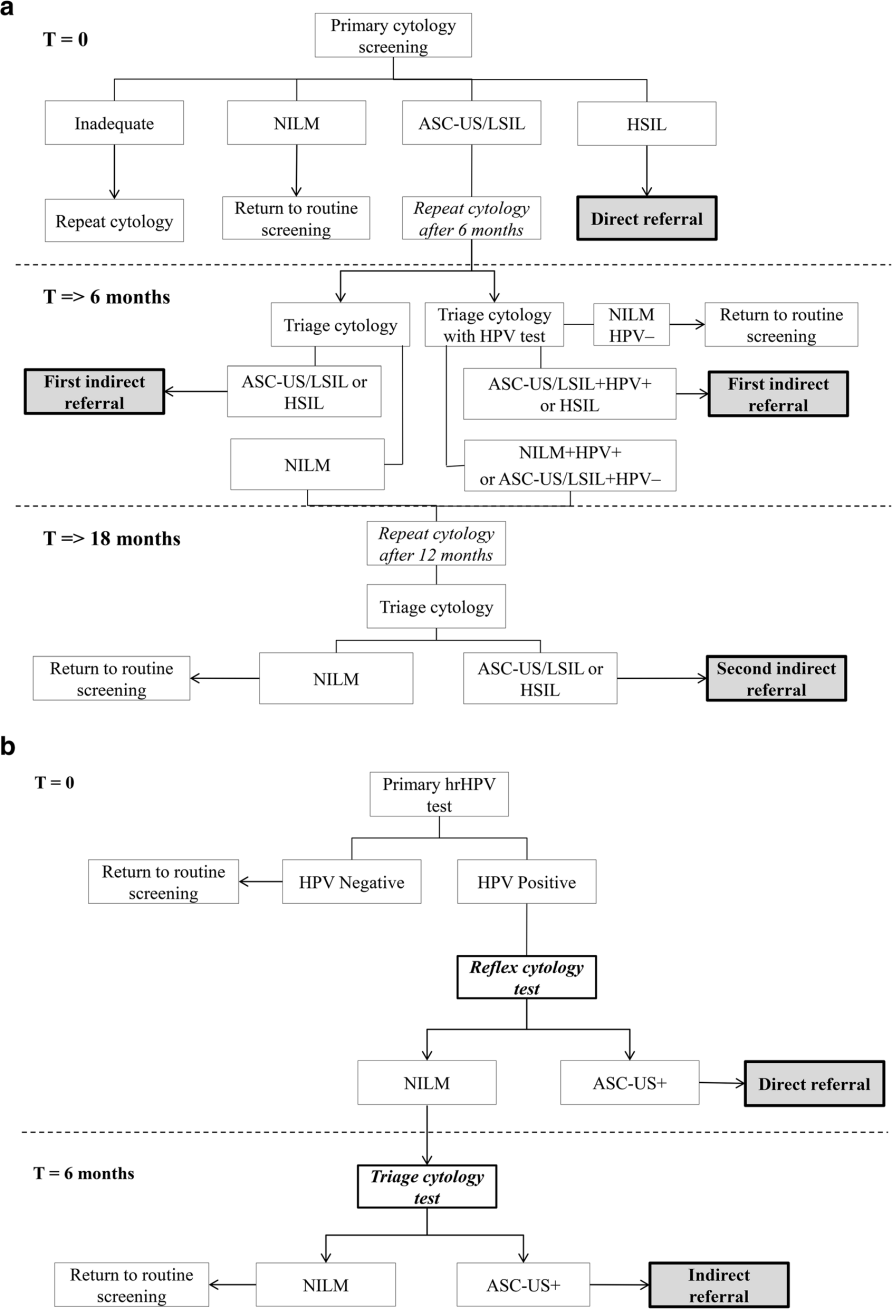
**a** Screening protocol of the cytology-based screening programme. **b** Screening protocol of the HPV-based screening programme. NILM negative for intraepithelial lesion or malignancy, ASC-US atypical squamous cells of undetermined significance, LSIL low-grade squamous intraepithelial lesion, HSIL high-grade squamous intraepithelial lesion

There were various referral pathways in the cytology-based programme, depending on the result of primary cytology screening (Fig. 1a). Direct referrals for colposcopy were given to women with high-grade cervical cytological abnormalities (high-grade squamous intraepithelial lesion (HSIL)) at primary screening. If women had low-grade cervical cytological abnormalities (atypical squamous cells of undetermined significance (ASC-US) or low-grade squamous intraepithelial lesion (LSIL)) at primary screening, they were advised to make an appointment with their GP after 6 months for a follow-up smear. For women advised to have a follow-up cytology at 6 months, hrHPV triage was used in some cases, depending on the policy of the laboratory performing the test. Referral advice was given to women at the 6-month screening who had the following result: (a) ASC-US or higher (when no hrHPV triage was performed) or, in the case of hrHPV triage, (b) ASC-US/LSIL and hrHPV-positive or (c) HSIL. Further repeat testing at 18 months was advised for women with cytology negative for intraepithelial lesion or malignancy (NILM) when no hrHPV triage was used or for NILM, hrHPV-positive results or ASC-US/LSIL, hrHPV-negative results. When hrHPV triage testing at 6 months was used, women were referred back for routine screening if they were hrHPV-negative and cytology-negative. All women with ASC-US+ cytology at 18 months were referred.

### The hrHPV-based Dutch cervical screening programme

Primary hrHPV screening was implemented in the Netherlands on 1 January 2017 (Fig. 1b), replacing the cytology-based programme. Women are invited to participate by their regional screening organisation every 5 years between the ages of 30 and 60, with some exceptions based on hrHPV positivity in the previous screening round; women with a negative hrHPV test result at age 40 or 50 are invited for screening after 10 years instead of 5 and women who test hrHPV-positive at age 60 are invited for final screening at age 65. Women who do not wish to have a cervical sample taken at their GP can request a self-sampling kit. If requested at primary invitation, women were sent the self-sampling kit approximately 4 months after the initial invitation letter. Non-responders received a reminder letter 4 months after the initial invitation, which also contained information about how to request the self-sampling kit. Women who requested the self-sampling kit after this reminder received it immediately. Reflex cytology was immediately performed on hrHPV-positive GP-collected samples. As cytology on self-sampled cervicovaginal material is unreliable [12, 13], women with an hrHPV-positive result on self-sampling were invited to have a cytological smear taken by their GP.

The referral algorithm in the hrHPV-based programme was simplified. HrHPV-positive women with cytological abnormalities (i.e. ASC-US or worse) were referred for colposcopy, while hrHPV-positive women with normal cytology were invited for repeat cytology testing after 6 months.

### Organisational and policy differences between the two programmes

In the Netherlands, there are five regional screening organisations responsible for the implementation of the screening programme. With the change from cytology-based to hrHPV-based screening, the policy for inviting women was changed, with the regional screening organisations sending all invitations in a standard manner; women were all invited after their birthday in the year they were eligible for invitation. In the cytology-based programme, invitations were sent by the regional screening organisation, GP practices or using a combined approach. The timing of the invitation also varied depending on which organisation sent the invitation; some invitations were sent at the start of the year that women would become eligible to participate, and some were sent after the women’s birthdate. The number of laboratories responsible for analysing primary screens from the programme was reduced from approximately 40 in the cytology-based programme to five in the hrHPV-based programme (one per region).

### hrHPV test in the new programme

Clinician-collected samples were collected in 20 ml ThinPrep medium (Hologic, Marlborough, MA, USA), transported and stored at room temperature until processed in the laboratory. The Evalyn® Brush (Rovers Medical Devices, Oss, the Netherlands) was used for self-sampling. The self-collected brushes were sent to the laboratories by regular mail. The brush of the self-sampling device was transferred into 20 ml of ThinPrep medium prior to hrHPV testing. All laboratories used the cobas® 4800 HPV test (Roche Diagnostics, Alameda, CA, USA) to test the clinician-collected and self-samples. The cobas® 4800 HPV test is a CE in vitro diagnostic (IVD) certified kit (for clinician-collected cervical scraps only) for use in combination with the cobas® 4800 system for nucleic acid extraction, PCR setup, real-time PCR amplification and result analysis. As part of the assay procedure, each sample was also tested for the presence of human cells by amplification of the human beta-globin gene. The clinical performance of the cobas® 4800 system has been validated using Dutch samples [14], and the Evalyn® Brush was compared with lavage self-sampling in a Dutch population and found to have equivalent performance [15]. All tests used in the hrHPV-based programme were selected through a tendering process.

### Study design and data source

This study is a longitudinal, retrospective population-based cohort study. We obtained results of primary screening tests and any associated follow-up from the Dutch nationwide network and registry of histo- and cytopathology (PALGA) for two cohorts. The cytology cohort consisted of women who participated in the cytology-based screening programme between 1 January 2015 and 31 March 2016 (maximum follow-up of 40 months). The hrHPV cohort consisted of women who participated between 1 January 2017 and 30 June 2018 in the hrHPV screening programme (maximum follow-up of almost 21 months). An inclusion period of 18 months was used for the hrHPV cohort to compensate for the phased implementation of the new programme (see Additional file 1).

All pathology laboratories in the Netherlands are linked to PALGA [11]. Identification of women is based on their birthdate and up to the first eight letters of their surname (maiden name is used for married women) and allows linkage of tests belonging to the same woman, enabling individual screening histories to be followed. For all primary and follow-up tests, the corresponding advice codes were analysed. Age was defined as the woman’s age at the time of the primary screening test, classified into 5-year age groups. Given differences in invitation policies between the two programmes, slightly different age ranges have been used for the hrHPV cohort and the cytology cohort (see Additional file 1).

### Data analysis

To compare the performance of the hrHPV-based screening programme with the cytology-based screening programme, we calculated indicators in three categories: *participation* (participation rate), *referral* (screen positivity rate, positive cytology amongst screen-positive women, referral rate from primary screening (direct referral), referral rate from follow-up smear (indirect referral) and total referral rate (direct and indirect referrals combined)) and *detection* (findings after referral per 1000 screened women, number of positive screen test results/number of referrals for colposcopy per detected CIN2+ or CIN3+ lesion).

The participation rate was defined by the number of primary screening tests divided by the number of women eligible for screening. The number of eligible women was estimated from the number of women in the Dutch population who would reach screening age in 2015 or 2017 (i.e. aged 29, 34, etc.) on 1 January 2015 for the cytology cohort and on 1 January 2017 for the hrHPV cohort. This data was obtained from Statistics Netherlands [16] and adjusted for the risk of having their cervix removed by hysterectomy [17].

Referrals were identified based on advice codes recorded in PALGA and could be direct or indirect (see Additional file 1). Overdiagnosis and false positive screening results are recognised harms of screening [18]. Screen positivity and referrals can lead to psychological distress [19, 20], and colposcopy itself can result in physical symptoms [21]. As such, we considered screen positivity and referral to be proxies for potential harms. To estimate the harms-benefits ratio of screening, we calculated the number of screen positives and number of referrals per detected CIN2+ and CIN3+ case. Detailed information about data definitions can be found in Additional file 1.

All analyses were performed using IBM SPSS Statistics 24. Chi-squared tests were performed to compare differences between proportions. *p* values of 0.05 or less were statistically significant.

## Results

### Participation

A total of 454,573 women eligible for screening invitation in 2017 participated in the hrHPV-based programme between 1 January 2017 and 30 June 2018 and 483,146 women eligible for screening invitation in 2015 participated in the cytology-based programme between 1 January 2015 and 31 March 2016. Women ranged in age from 29 to 61 years.

**Fig. 2:**
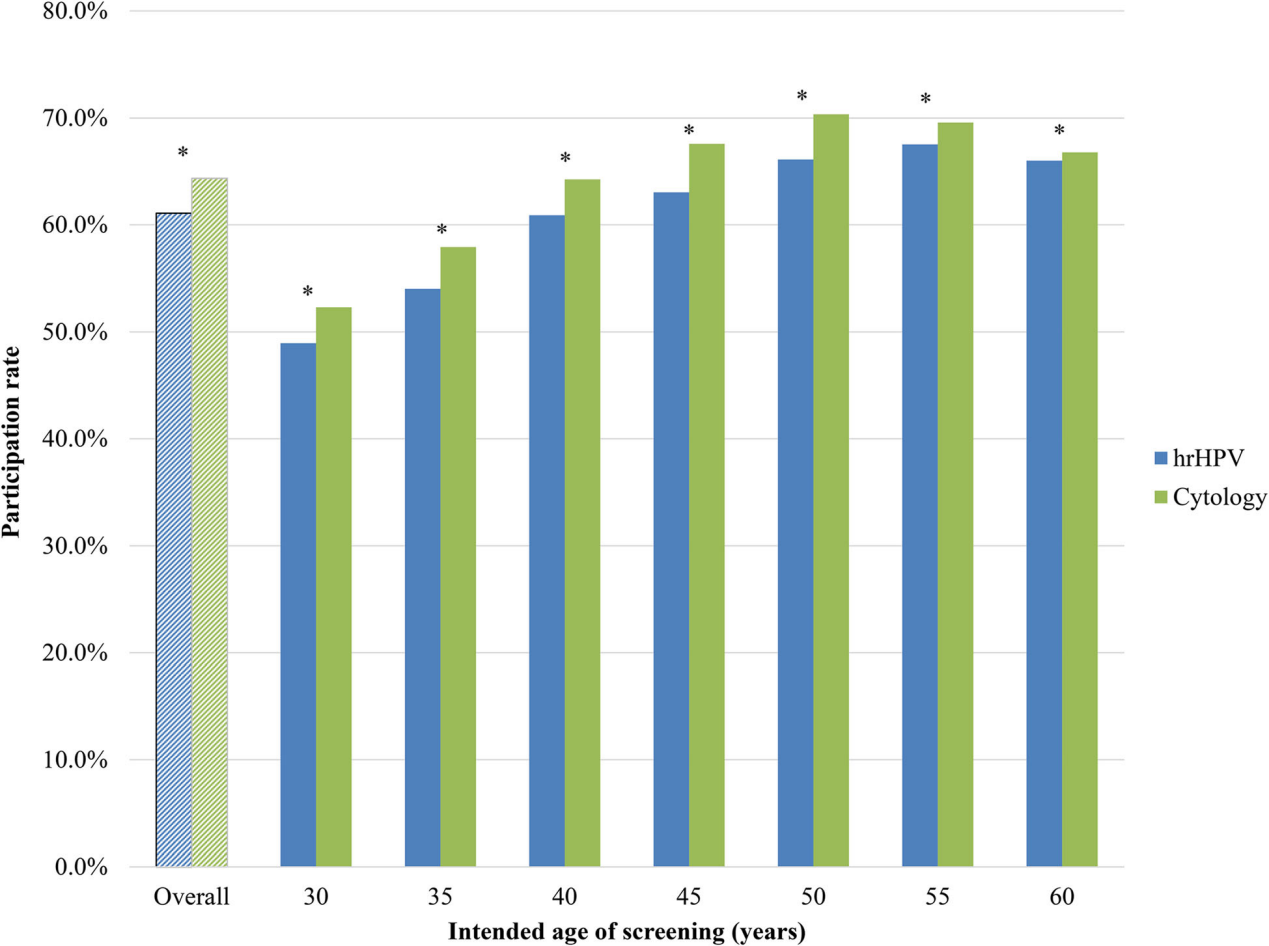
Participation rate in hrHPV-based screening (2017) and in cytology-based screening (2015) by age. 454,573 women participated in the hrHPV-based screening programme, and 483,146 women participated in the cytology-based screening programme. N.B. Please refer to Additional file 1 for a comprehensive explanation of age group criteria. *Pearson’s chi-square test significantly different between test types (*p* < 0.001)

Figure 2 shows that the overall participation rate in the hrHPV-based programme in 2017 was significantly lower than that in the cytology-based screening programme in 2015 (64% in 2015 compared with 61% in 2017; *p* < 0.001). The participation rate in the hrHPV-based programme was lower in all age groups. The biggest difference was found in age group 45–49 years (68% in 2015 compared with 63% in 2017; *p* < 0.001). Differences in participation rates were statistically significant for all age groups (*p* < 0.001).

The percentage of inadequate cytology smears recorded at primary screening as a proportion of all primary screening reduced from 1.6% in 2015 to 0.1% in 2017 (*p* < 0.001).

**Fig. 3:**
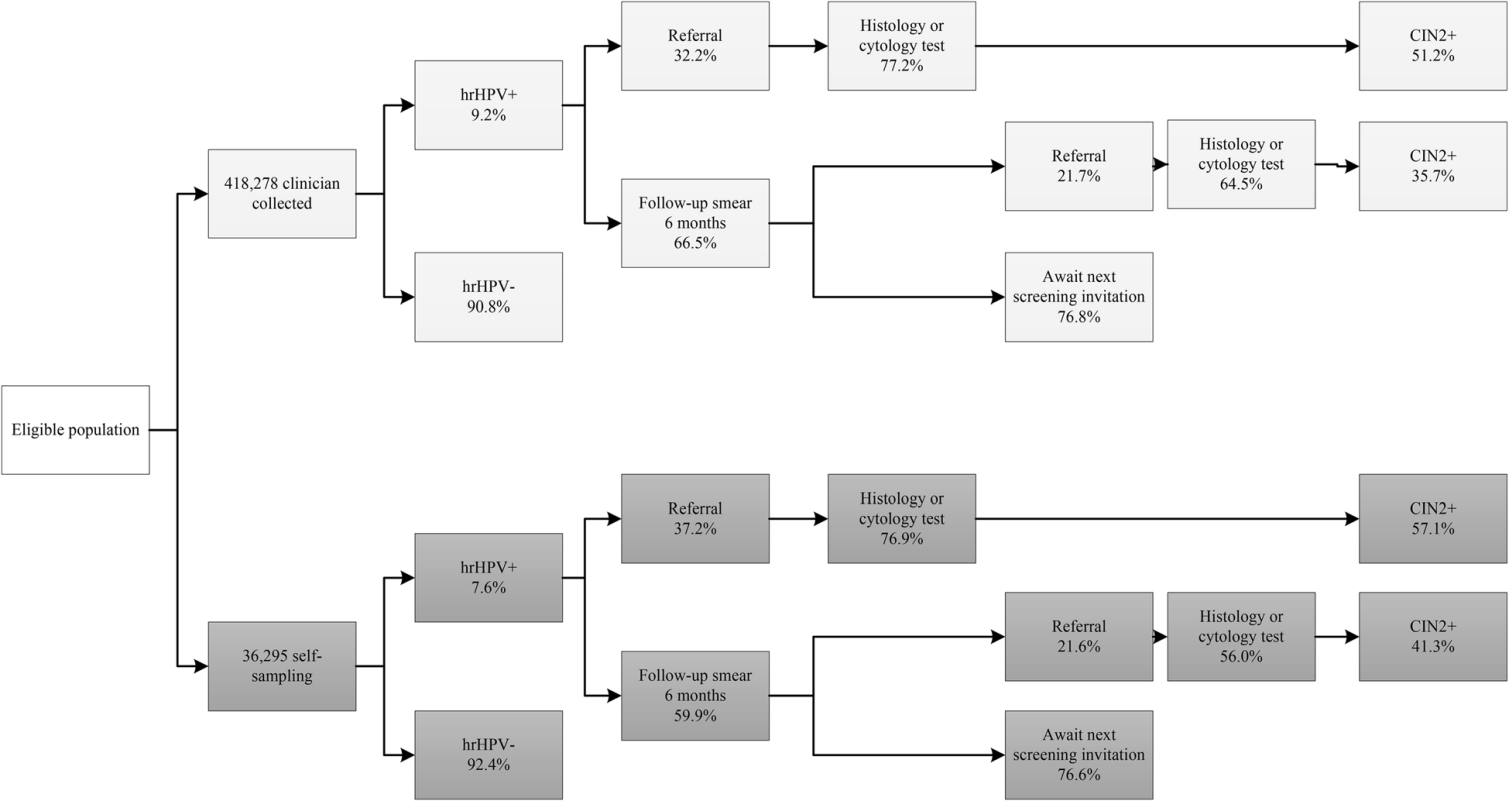
Flowchart of participation, referral and detection within the new hrHPV-based screening programme, 2017 cohort. Pearson’s chi-square test significantly different for hrHPV positivity, direct referral rates and follow-up smear (*p* < 0.001) and CIN2+ detection rates from direct referral (*p* = 0.002) between clinician-collected and self-sampling. Pearson’s chi-square test not significantly different for proportions of histology or cytology tests (from direct referral, *p* = 0.805; from indirect referral, *p* = 0.042), indirect referral rate (*p* = 0.974), proportions with recommendation to await next screening invitation (*p* = 0.884), CIN2+ detection rates from indirect referral (*p* = 0.319) between clinician-collected and self-sampling. N.B. Sum of advice after screening will not be 100% due to a proportion of screens with repeat cytology due to inadequate cytology quality or loss to follow-up (self-sampling arm only). Cytology was assessed in 90.1% of hrHPV-positive cases in the self-sampling arm. Repeat cytology because of inadequate cytology quality after a positive screen result was recommended in 1.3% of clinician-collected cases and 1.6% of self-sampling cases with cytology (1.3% of self-sampling cases had other recommendations). Repeat cytology because of inadequate cytology quality in a follow-up smear at 6 months was recommended in 1.5% of clinician-collected cases and 1.8% of self-sampling cases

Of all women participating in the hrHPV-based programme, 8% used the self-sampling kit (i.e. 36,295 self-sampled compared with 418,278 clinician-collected) (Fig. 3).

### Referral

**Fig. 4:**
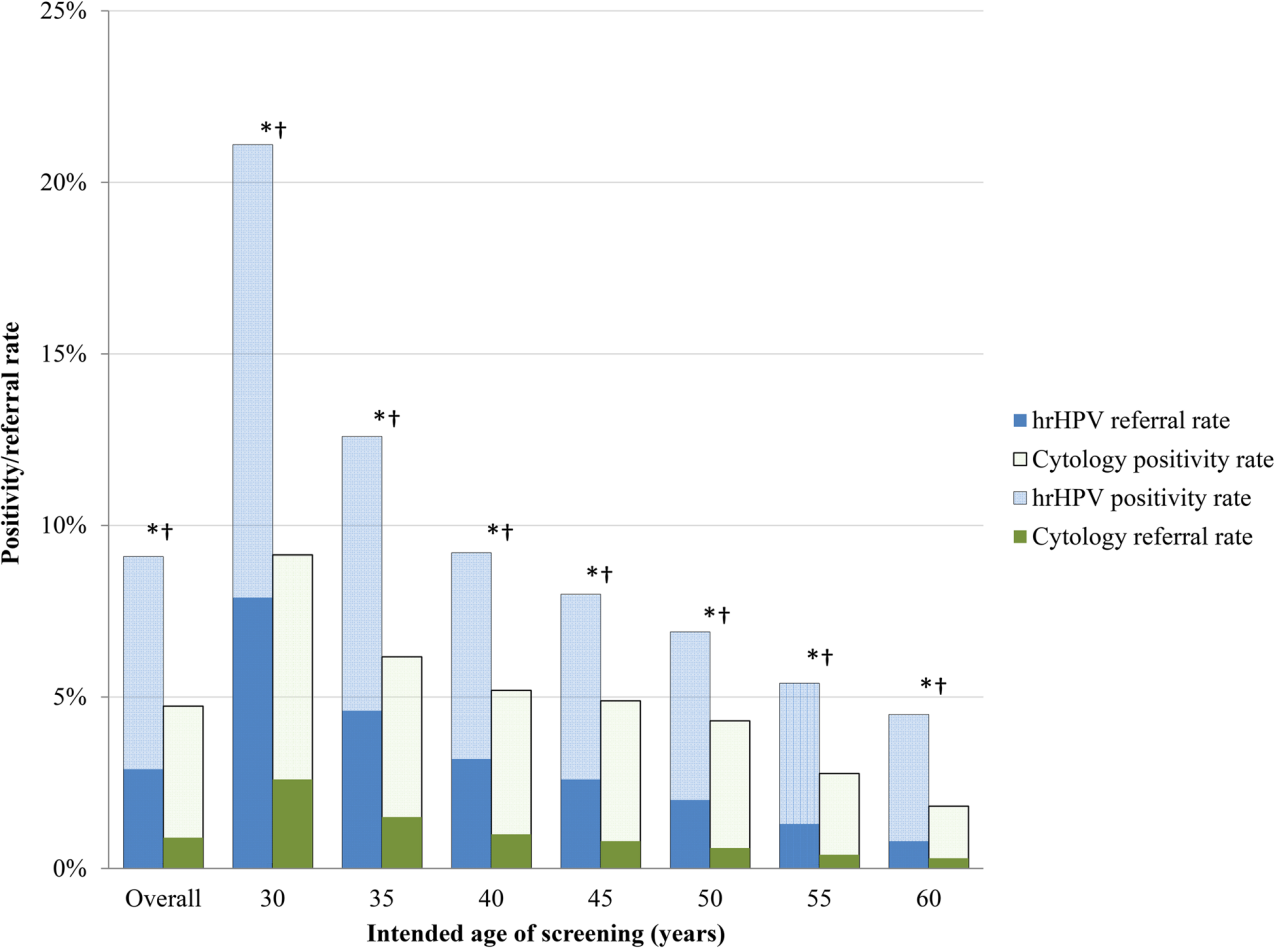
Screen positivity and direct referral rates by screening programme and age. Cytology-based screening results are based on the 2015 screening cohort, and hrHPV-based screening results are based on the 2017 screening cohort. Screen positivity in the hrHPV-based screening programme is hrHPV-positive, irrespective of reflex cytology results. 454,573 women participated in the hrHPV-based screening programme, and 483,146 women participated in the cytology-based screening programme. N.B. Please refer to Additional file 1 for a comprehensive explanation of age group criteria. *Pearson’s chi-square test significantly different for screen positivity rates between test types (*p* < 0.001). †Pearson’s chi-square test significantly different for referral rates between test types (*p* < 0.001)

Figure 4 shows that the proportion of women with a positive screen test was significantly higher in the hrHPV-based programme than in the cytology-based programme (increased from 5% in 2015 to 9% in 2017; *p* < 0.001). Related to this, we found that the proportion of women referred to the gynaecologist also significantly increased (from 1% in the cytology-based programme to 3% in the hrHPV-based programme; *p* < 0.001). The increases in positive screen tests and in the referral rate were largest in women aged 30–34 years, where the proportion of positive screen tests increased from 9% in the cytology-based programme to 21% in the hrHPV-based programme (*p* < 0.001) and the referral rate increased from 3% to 8% (*p* < 0.001).

In the hrHPV-based programme, we found a significantly higher hrHPV positivity rate in clinician-collected than in self-collected samples (9.2% vs 7.6%; *p* < 0.001). In addition, amongst hrHPV-positive women, more women had a cytological abnormality after self-sampling than clinician-collected sampling (37.2% vs 32.2%; *p* < 0.001) (Fig. 3).

### Detection

**Fig. 5:**
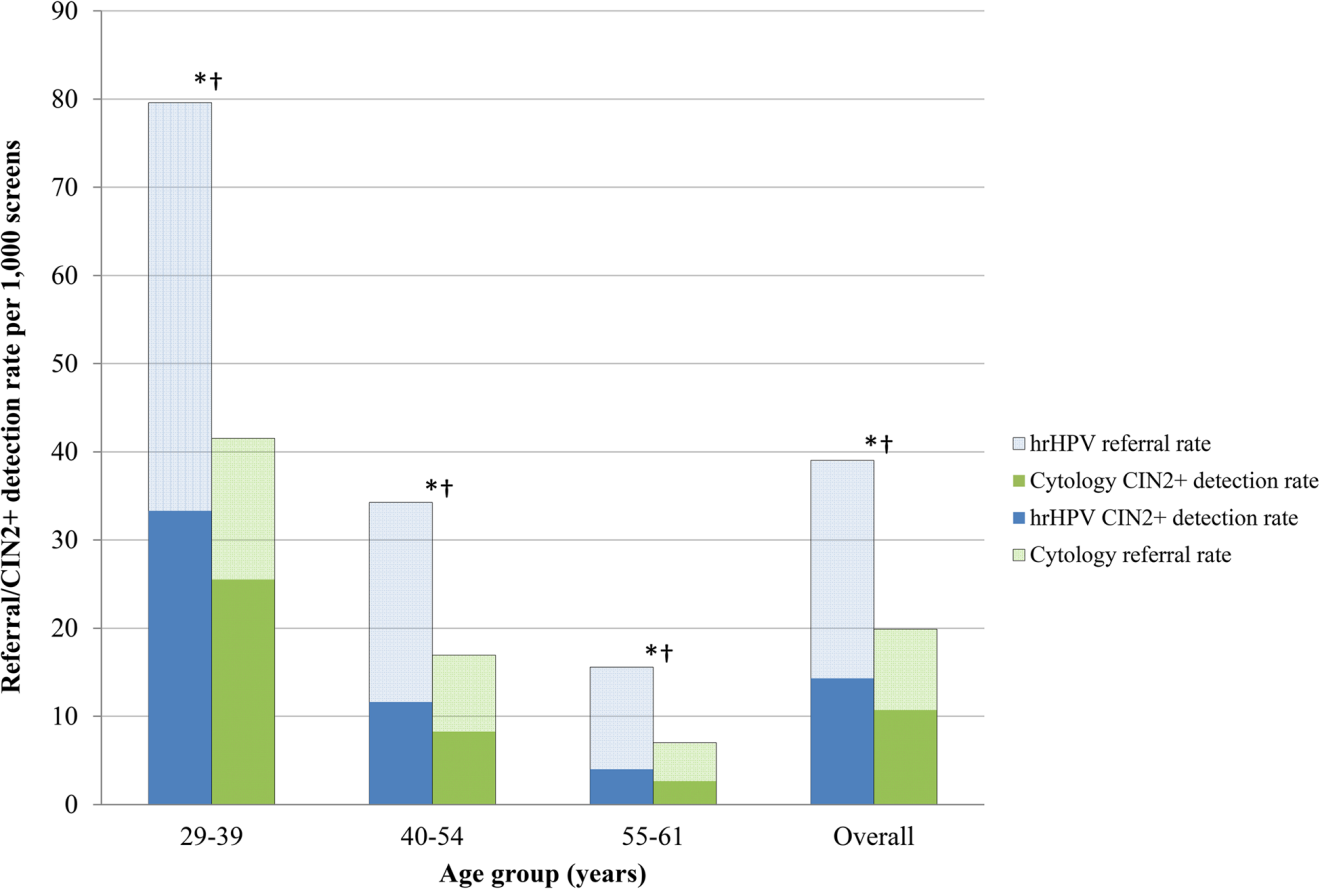
Total referral and CIN2+ detection rates in all screened women by screening programme and age. Cytology-based screening results are based on the 2015 screening cohort, and hrHPV-based screening results are based on the 2017 screening cohort. 454,573 women participated in the hrHPV-based screening programme, and 483,146 women participated in the cytology-based screening programme. Referral rates include direct and indirect referrals. N.B. Please refer to Additional file 1 for a comprehensive explanation of age group criteria. *Pearson’s chi-square test significantly different for referral rates between test types (*p* < 0.001). †Pearson’s chi-square test significantly different for CIN2+ detection rates between test types (*p* < 0.001)

Figure 5 shows, per 1000 women screened, the total number of referrals (both direct and indirect) to the gynaecologist and the number of CIN2+ lesions detected after referral. The number of referrals increased from 20 to 39 per 1000 women screened, and the CIN2+ detection rate increased from 11 to 14 per 1000 women screened (*p* < 0.001). Overall, the referral rate doubled and the CIN2+ detection rate increased by 34% (*p* < 0.001). For the youngest age group, the referral rate increased by 92% (*p* < 0.001) and the CIN2+ detection rate by 30% (*p* < 0.001).

Cytology or histology was performed in 77% of women directly referred to the gynaecologist in the hrHPV-based programme (Fig. 3). In the remaining 23%, only colposcopy was performed after referral or women were lost to follow-up. In the case of indirect referrals, in 64.5% of clinician-collected or 56.0% of self-sampling (*p* = 0.974), cytology or histology was performed. The CIN2+ detection rate after cytology or histology varied across the four different groups in the hrHPV-based programme: from 35.7% in indirect referred women after a clinician collected sample to 57.1% in direct referred women after self-sampling (Fig. 3).

Table 1 shows the different findings after direct and indirect referrals for the hrHPV-based and cytology-based programmes. We found that in the hrHPV-based programme after referral, approximately 2.2 times more clinically irrelevant findings were found (i.e. ‘cytology only’, ‘no dysplasia’ or CIN1), compared with approximately 1.3 times more clinically relevant findings (i.e. CIN2, CIN3 and cancer).

**Table 1:** Findings after referrals for colposcopy by screening programme, referral type and age, per 1000 women screened

Rate per 1000 screened women	HPV								Cytology							
	Direct**				Indirect**				Direct**				Indirect**			
	Overall	29–39	40–54	55–61	Overall	29–39	40–54	55–61	Overall	29–39	40–54	55–61	Overall	29–39	40–54	55–61
No follow-up with cytology or histology test*	6.0	12.5	5.2	2.3	3.4	6.0	3.1	1.9	0.5	0.8	0.4	0.4	2.3	4.0	2.3	0.9
Cytology only	0.7	1.1	0.6	0.4	0.1	0.3	0.1	0.1	0.2	0.2	0.2	0.1	0.2	0.3	0.2	0.1
No dysplasia	3.9	6.5	3.9	2.0	1.6	2.6	1.5	1.0	0.6	0.8	0.5	0.5	1.8	2.8	1.8	0.9
CIN1	6.3	12.8	5.6	2.4	2.1	3.8	2.1	1.0	0.9	1.7	0.8	0.4	3.0	5.8	3.0	0.9
CIN2	4.7	10.6	4.0	1.3	1.2	2.2	1.1	0.5	2.0	4.7	1.5	0.6	2.3	4.8	2.1	0.7
CIN3	6.9	17.2	5.3	1.7	1.1	2.4	0.8	0.5	4.9	12.5	3.5	1.0	1.4	3.5	1.2	0.3
Cancer	0.4	0.9	0.4	0.1	0.0	0.0	0.0	0.0	0.3	0.6	0.3	0.1	0.0	0.1	0.0	0.0

N.B. Cases with a histological record that is coded as ‘no diagnosis’ (average of 1.2% of total cases) are included in the denominator but not presented in the table. Please refer to Additional file 1 for a comprehensive explanation of age group criteria

*These women are referred for colposcopy, but no follow-up examination has been registered in PALGA. These women are either lost to follow-up or only colposcopy is performed

**Pearson’s chi-square test significantly different for the distribution of outcomes between test types (*p* < 0.001)

### Harms versus benefits

Table 2 shows the number of positive screen tests and number of referrals (i.e. ‘harms’) per CIN2+ and CIN3+ lesion detected (i.e. ‘benefits’) in one screening round, for both the hrHPV-based and cytology-based screening programmes. We found that in the new programme, the harms per benefit increased by approximately 45% in one screening round for CIN2+ lesions and by 51% for CIN3+ lesions. For example, to detect one CIN3+ lesion in the cytology-based programme, 3.0 women were referred, compared to 4.6 in the hrHPV-based programme. This difference was mostly due to the increase in referrals of hrHPV-positive screens with ASC-US/LSIL cytology in the hrHPV-based programme, which stemmed from a national policy change to refer, rather than observe, hrHPV-positive screens with ASC-US/LSIL results.

**Table 2:** Number of positive screen tests and number of referrals per detected CIN2+ or CIN3+ lesion

		Cytology	HPV	Difference per round (%)
**Positive screens**				
Total*				
Number of positives needed to detect one	CIN2+	4.4	6.3	44
	CIN3+	7.2	10.8	50
**Referrals**				
Total*				
Number of referrals needed to detect one	CIN2+	1.9	2.7	47
	CIN3+	3.0	4.6	53
**HSIL**				
Number of referrals needed to detect one	CIN2+	1.3	1.3	−2
	CIN3+	1.8	1.8	−2
**ASC-US/LSIL**				
Number of referrals needed to detect one	CIN2+	3.0	4.7	57
	CIN3+	7.5	12.0	60

N.B. Triage algorithms for ASC-US/LSIL screens differ between the cytology-based and hrHPV-based programmes; in the hrHPV-based programme, all hrHPV-positive, ASC-US/LSIL screens are directly referred, whereas in the cytology-based programme, ASC-US/LSIL screens were triaged for repeat cytology after 6 months

*Total includes all positive hrHPV tests irrespective of the reflex cytology result (includes hrHPV-positive screens with reflex cytology of NILM, inadequate or missing)

## Discussion

### Main findings

The nationwide implementation of primary high-risk HPV DNA screening in the Netherlands has been successful, with the programme now fully implemented and results generally as expected, apart from a lower than anticipated participation rate. In the first year, we observed a participation rate of 61%, which was lower than observed in the previous cytology-based programme (64%). Screen positivity was higher in the hrHPV-based programme. The cytology programme recommended observation of ASC-US/LSIL results, while the hrHPV-based programme recommended colposcopic referral for hrHPV-positive, ASC-US/LSIL results. As expected, this increased both the number of colposcopic referrals and CIN2+ lesions detected.

### Factors influencing participation rates

The introduction of self-sampling had been expected to increase participation, as a previous Dutch study (PROHTECT) found that screening non-attenders who were offered self-sampling were more likely to be screened than non-attenders [22]. While 8% of screened women used self-sampling, this did not increase overall participation, suggesting that switching is occurring. Information about switching was not publicly reported in the 2017 official monitoring report [23], and further research is needed into the characteristics of women who choose for self-sampling to provide reliable estimates of this indicator. One important difference between PROHTECT and the real-world implementation was that women needed to opt in to self-sampling in the screening programme. Secondly, the 4-month waiting period for the self-sampling kit may have delayed uptake of screening amongst women who opted in. The self-sampling kit may be used by women who find it more convenient than attending the GP; one of the main reasons identified in a Dutch study for using a self-sampling kit [24]. Finally, although self-sampling is generally acceptable to women [12], 23% of self-sampling kits requested by the 2017 cohort have not yet been returned (as of December 2018; personal communication, RIVM, 21 December 2018). Although the return of these kits would not have a large effect on overall participation, the reasons for not returning them should be further investigated.

Organisational factors, such as the phased roll-out of the new programme and changes in the invitation process, may also have resulted in lower participation. Due to the phased roll-out of the new programme over the first quarter of 2017, women had less time to take up their screening invitations compared with the cytology-based programme, although we still observed a lower participation rate when calculating it based on 18 months of data. If the phased implementation is the cause of lower participation, we would expect participation to increase in the coming months. In the cytology-based programme, GP practices could invite patients for screening, rather than women receiving an invitation from the regional screening organisation. Women who received invitations sent from GP practices were more likely to participate in the cytology-based programme than women who received invitations from screening organisations [25]. Discontinuing the involvement of GP practices in the invitation and reminder process may have led to a decline in participation, as invitations are now sent from organisations that may be unfamiliar to women; this needs further investigation.

### Comparison with other studies

The hrHPV positivity rate was higher than anticipated at 9.1%, as a previous population-based Dutch study (DuSC) found a hrHPV positivity rate of 8% amongst women of screening age [26]. This difference may be explained by differences in sociodemographic characteristics of women participating in the programme overall and the women included in DuSC. It could also be that there has been an increase in the incidence of hrHPV infections over time. The higher than expected hrHPV positivity rate may explain differences between the estimated referral rate of 3.4% (based on modelling) [6] and the observed referral rate of 3.9%.We found 48.2% CIN2+ detection in all women with histologically confirmed diagnosis, which was higher than the rate predicted by modelling (45%), which may be due to differences in the assumed test characteristics and the real-world performance of the hrHPV test [6].

One surprising finding was that hrHPV positivity was lower in self-samples than in the clinician-collected samples, contrary to previous Dutch studies. One population-based study found higher hrHPV positivity in self-samples than in clinician-collected samples [12], and one randomised non-inferiority trial (IMPROVE) found equivalent hrHPV positivity between the two test types, although IMPROVE used a different clinician-collected test than is used in the screening programme [27]. Despite this, we found higher CIN2+ detection in self-sampling than in clinician-collected sampling. This may indicate that the self-sampling test has a higher CIN2+ specificity than the clinician-collected test, in contrast to results from IMPROVE, which reported CIN2+ specificity of the self-test was non-inferior (relative accuracy of 1.00) [27]. Further analysis of the self-sampling kit within the screening programme is needed, controlling for background risk and population factors.

### Triage of hrHPV-positive women

A higher CIN2+ detection rate was found in the hrHPV programme than in the cytology-based programme. This was expected based on the results of four large randomised trials of HPV screening [1]. However, in the new hrHPV screening programme, more referrals per screening round were needed to detect one CIN2+ lesion compared with cytology-based screening, mainly due to an increase in the number of referrals amongst women with ASC-US/LSIL cytology. This increase potentially leads to more harms for women, including anxiety for women unnecessarily referred [19] or potential overtreatment of low-grade lesions. Therefore, optimising triage to reduce unnecessary referrals should be a priority. Different triage strategies for hrHPV-positive screens have been proposed, including (but not limited to) p16/Ki67 dual staining, hrHPV genotyping, methylation, HPV E6 protein assays or combinations of these strategies [28]. Risk-based management could also be explored, in which risk factors (such as a woman’s screening history) are taken into account when triaging hrHPV-positive, ASC-US primary screens [29]. The performance of additional triage tests in the Dutch setting as well as the feasibility of implementation, any impacts on programme cost-effectiveness and the balance of harms versus benefits of the screening programme need to be considered prior to changing the triage algorithm. The harms-benefits ratio of the old cytology-based programme was considered acceptable in the Netherlands, and while in one round of screening the hrHPV-based screening programme had a more unfavourable balance, reducing the number of total screening rounds in the hrHPV-based programme (from seven to five for many women) will result in similar overall lifetime harms-benefits ratio to that of the cytology-based programme.

### International comparisons

In several countries, hrHPV-based screening has been implemented, but published results are only available from Italy and Turkey. In Italy, HPV-based screening was implemented in 2012 in 19 screening programmes across ten regions. The direct referral rate from the Italian programme was comparable with the Dutch programme at 2.9% [10]. In 2014, primary HPV screening was implemented in Turkey; however, a direct comparison of results is difficult due to a low participation rate (36.5%) and incomplete histological follow-up data [9]. Neither study compared hrHPV-based screening with cytology-based screening. In general, the quality of a cytology-based programme influences such a comparison. In the Netherlands, the quality of the cytology-based programme was consistently high, with low rates of unsatisfactory smears and a high positive predictive value for CIN2+ lesions compared with other European countries [30]. In a country with a less highly performing cytology programme, the incremental effects of HPV-based screening versus cytology-based screening would be different.

### Future implications for hrHPV screening in partly vaccinated cohorts

Given the increased sensitivity of hrHPV testing for CIN2+ lesions, detection rates are expected to be higher in the first round, as both prevalent and incident lesions are detected. As the programme reaches a steady state, and fewer prevalent lesions are detected, we expect that detection of CIN3+ lesions will decrease, as seen in the POBASCAM trial [31]. Therefore, it will be necessary to compare results from the first and subsequent screening rounds. In the Netherlands, hrHPV vaccination was offered in a catch-up programme to girls aged 13 to 16 years in 2009, meaning the first cohort of partly vaccinated women will be eligible for screening in 2023. This may necessitate changes to the programme, due to an anticipated reduction in HPV16/18 infections. Modelling has shown that with herd immunity levels greater than 50%, a reduction in the number of screening rounds may need to be considered to maintain programme cost-effectiveness in the Netherlands [32]. Finally, for full evaluation of the new screening programme, calculation of interval cancer incidence is essential to approximate the sensitivity of one screening round. Women are at highest risk of an interval cancer diagnosis 4 to 6 years after a negative screen [33], as the screening interval is 5 years. As such, the first opportunity for comparison of this indicator will come 5 years after the implementation of hrHPV-based screening.

### Strengths and limitations of this study

This is the first study to report the results of the nationwide implementation of a hrHPV-based screening using prospectively collected cyto- and histopathological data. We have been able to compare this reliably with the previous cytology-based programme due to the nationwide coverage of PALGA. The large number of screens included in our study has allowed us to make statistically robust comparisons between indicators of the two programmes. Our study has some limitations. The follow-up time included in our study was shorter for the hrHPV-based programme than for the cytology-based programme, as the hrHPV-based programme was implemented more recently. We are unable to analyse characteristics of non-attenders to the programme, as characteristics of these women are not captured by PALGA. We are also unable to differentiate loss to follow-up after referral for colposcopy from cases where women attended colposcopy, but no cytology or histological diagnostic test was performed. This information is unavailable for both the hrHPV-based programme and the cytology-based programme. As such, we cannot investigate whether adherence to referral advice has changed over time. Furthermore, compliance to referral, used to differentiate cytology only and no follow-up with cytology or histology in Table 1, may have been underestimated for hrHPV screening due to the shorter follow-up time for the hrHPV-based programme; however, without data on colposcopies, the extent of this underestimation is unknown. The identifier used in PALGA to link records is non-unique (based on the first eight letters of a woman’s surname and her date of birth). This means that records from multiple women could be linked to one identifier (called an administrative fusion). It is unlikely that there is a difference in the number of administrative fusions between the two programmes, and therefore, we expect that this has not influenced our results. Finally, because the cytology-based programme recommended observation of ASC-US/LSIL results, while the hrHPV-based programme recommended colposcopic referral for hrHPV-positive, ASC-US/LSIL results, distinguishing the relative impact of the hrHPV test itself versus the lower threshold for referral on both unnecessary testing and CIN2+ detection is difficult.

## Conclusions

This is the first time that results of nationwide implementation of hrHPV-based screening have been reported using high-quality data with extended follow-up. Our results show implementation of the hrHPV-based programme has been successful. However, the lower participation rate in the hrHPV-based programme needs to be investigated further to ensure that the screening programme remains effective and efficient. Detection of CIN2+ lesions was higher in the hrHPV-based programme at the cost of more unnecessary referrals. Careful consideration needs to be given to potentially changing triage of hrHPV-positive screens to reduce unnecessary referrals. Ongoing monitoring of the hrHPV-based programme is essential to ensure that a reasonable balance of benefits and harms continues to be achieved.

## Supplementary information

**Additional file 1. **Detailed description of methods for calculating results*.* **Table A1.** Age groupings used in analysis by programme type. **Table A2.** Calculation of the indicators shown in Figure 2 for participation, referral and detection within the new hrHPV-based screening programme, 2017 cohort. **Table A3.** Calculation of the indicators for participation, referral and detection in the old cytology-based screening programme, cohort 2015, and within the new hrHPV-based screening programme, 2017 cohort.

## Data Availability

The data that support the findings of this study are available on request from PALGA, the nationwide network and registry of histo- and cytopathology in the Netherlands, but restrictions apply to the availability of these data.

## Abbreviations

ASC-US: Atypical squamous cells of undetermined significance
CIN: Cervical intraepithelial neoplasia
GP: General practitioner
hrHPV: High-risk human papillomavirus
HSIL: High-grade squamous intraepithelial lesion
LSIL: Low-grade squamous intraepithelial lesion
PALGA: Dutch nationwide network and registry of histo- and cytopathology
NILM: Negative for intraepithelial lesion or malignancy
RIVM: Rijksinstituut voor Volksgezondheid en Milieu (Dutch National Institute for Public Health and the Environment)
