# Supplementary material for: Introduction of primary screening using high-risk HPV DNA detection in the Dutch cervical cancer screening programme: a population-based cohort study
Source: BMC Med. 2019 Dec 11;17:228. doi: 10.1186/s12916-019-1460-0 (PMC6907114; doi:10.1186/s12916-019-1460-0)
Supplement: Supplementary file 1 — Additional file 1. Detailed description of methods for calculating results. Table A1. Age groupings used in analysis by programme type. Table A2. Calculation of the indicators shown in Figure 2 for participation, referral and detection within the new hrHPV-based screening programme, 2017 cohort. Table A3. Calculation of the indicators for participation, referral and detection in the old cytology-based screening programme, cohort 2015, and within the new hrHPV-based screening programme, 2017 cohort. [file 12916_2019_1460_MOESM1_ESM.docx]

**Additional file 1: Detailed description of methods for calculating results**

*Supplement to: Aitken CA, van Agt HME, Siebers AG et al. Introduction of primary screening using high-risk HPV DNA detection in the Dutch cervical cancer screening programme: a population-based cohort study*

To calculate results for our study, we used extracts of all cervical cytology and histology records from the nationwide network and registry of histo- and cytopathology in the Netherlands (PALGA). Primary screening tests were selected from 1 January 2015 to 31 March 2016 for the cytology cohort and from 1 January 2017 to 30 June 2018 for the hrHPV cohort. We chose not to select 1 January 2016 to 31 March 2017 as the comparison period for cytology, due to an overlap with the new programme. The maximum follow-up time for the cytology cohort was 40 months and four days for screens taken on 1 January 2015 (end date of dataset: 4 May 2018) and the maximum follow-up time for the hrHPV cohort was 20 months and 28 days for screens taken on 1 January 2017 (end date of dataset: 28 September 2018).

*Ages included in analysis*

Due to changes in the organisation of invitations, grouping of age is slightly different between the old cytology-based programme and the new hrHPV-based programme. In the cytology-based programme, women could be invited at different dates in the year that they were eligible for screening; this could be at the start of the year they were eligible for screening, on their birth date or other time during the year that they were eligible for screening. Invitations could also be sent by different organisations (the regional screening organisation, the woman’s GP or a combined approach). In the hrHPV-based programme, women are sent an invitation letter on their birth date from their regional screening organisation. Due to these differences, many women aged 29, 34, 39, 44, 49, 54 and 59 had primary screening in the cytology cohort between 1 January 2015 and 31 March 2016. The age categories outlined in Table A1 for the cytology cohort have been used for many years to categorise age in the annual screening programme Monitor published by the Dutch National Institute for Public Health and the Environment. For this reason, we have used these age groupings for the cytology cohort in our study.

Table A1 shows how age group was defined in our analysis. A very small number of 29 year olds (36 in total) had a primary screening test recorded in the hrHPV-based programme between 1 January 2017 and 30 June 2018; these women were included in the 30 years age group.

***Table A1:*** *Age groupings used in analysis by programme type.*

|  | **Age groupings used in monitoring reporting** | |
| --- | --- | --- |
| **Label used in this study** | **Cytology-based programme** | **hrHPV-based programme** |
| 30 years | 29-33 years | 29*-34 years |
| 35 years | 34-38 years | 35-39 years |
| 40 years | 39-43 years | 40-44 years |
| 45 years | 44-48 years | 45-49 years |
| 50 years | 49-53 years | 50-54 years |
| 55 years | 54-58 years | 55-59 years |
| 60 years | 59-63 years** | 60-64 years** |

* 36 women aged 29 years had screening registered as part of the hrHPV-based screening programme in 2017.

** The maximum age of women included in this study was 61 years, however, five-year age categories are used in the Monitoring reports for the cervical cancer screening programme.

*Participation*

In the cytology-based programme, participants were defined by the number of screening test results from clinician-based sampling, performed between 1 January 2015 and 31 March 2016. The eligible population was based on the number of women who would reach screening age in 2015 in the Dutch population on 1 January 2015 (i.e. aged 29, 34, etc.), adjusted for the risk of having their cervix removed by hysterectomy.

In the hrHPV-based programme, participants were defined by the number of screening test results from clinician-based sampling or self-sampling, performed between 1 January 2017 and 30 June 2018. The eligible population was based on the number of women who would reach screening age in 2017 in the Dutch population on 1 January 2017 (i.e. aged 29, 34, etc.), adjusted for the risk of having their cervix removed by hysterectomy.

*Referral*

Table A2 and Table A3 shows the definitions used to calculate the direct and indirect referral rates in the cytology-based programme and hrHPV-based programme.

In the cytology-based programme, there were two triages for repeat cytology (first indirect at 6 months and second indirect at 12 months; see Figure 1a). Therefore, the indirect referral rate combines first and second indirect referrals. This rate was calculated amongst women who complied to the advice for repeat cytology within 365 days from the primary screening test for 6 months repeat cytology, and within 630 days from the 6 months cytology test for 12 months repeat cytology.

In the hrHPV-based programme, indirect referral rates were calculated in women who complied to the advice for repeat cytology within 365 days from of the date of primary screening.

*Detection*

Table A2 and Table A3 shows the definitions used to calculate the detection rates in the cytology-based programme and hrHPV-based programme.

Detection rates were calculated in women who were referred to the gynaecologist (due to their result on the screening test or their result from repeat cytology) and complied to the referral advice. In Figure 2, all detection rates are calculated amongst women who complied with referral advice within 150 days of a primary screening or follow-up test. In Tables 1 and 2, compliance within 150 days was only used to define the ‘no follow-up with cytology or histology test’ and ‘cytology only’ groups.

Women who complied to referral (i.e. they had an examination after 150 days from the referral advice, either from screening test of repeat cytology) but did not have a histology result were assumed to have had a cytology test only. The most severe histological diagnosis that was recorded within the episode of screening was used to categorise histology results.

Colposcopies without a histology or cytology test were not registered in the PALGA database. Referred women who did not comply, according to the definition, may therefore consist of women who are lost to follow-up or women who had colposcopy without histology or cytology.

*Harms vs. benefits*

To estimate the harms-benefits ratio of screening, we calculated the number of screen positives per detected CIN2+ and CIN3+ case and number of referrals per detected CIN2+ and CIN3+ case.

***Table A2:*** *Calculation of the indicators shown in Figure 2 for participation, referral and detection within the new hrHPV-based screening programme, 2017 cohort.*

| Indicator | Numerator | Denominator |
| --- | --- | --- |
| **Participation** |  |  |
| Participation rate | *Participants*, i.e. number of screening tests from clinician-based sampling or self-sampling, performed between 1 January 2017 and 30 June 2018. | *Eligible population*, i.e. number of women at screening ages in the Dutch population on 1 January 2017, adjusted for the risk of having their cervix removed by hysterectomy. |
| **Referral** |  |  |
| hrHPV positivity | *Screen positives*, i.e. number of hrHPV positive screening tests | Participants |
| Cytology assessments amongst screen positives | Number of hrHPV positives with a cytology result | Screen positives |
| Referral rate from primary screening (direct referral) | *Direct referrals*, i.e. number of hrHPV positive screening tests with ASC-US+ cytology result | Number of screen positives with cytology assessment |
| Advice for follow-up smear after 6 months | *Triage cytology advice,* i.e. number of screen positives with NILM cytology | Number of screen positives with cytology assessment |
| Referral rate from follow-up smear (indirect referral) | *Indirect referrals*, i.e. number of follow-up smears with an ASC-US+ cytology result | Number of triage cytology performed within 365 days from the screening test. |
| **Detection** |  |  |
| Histology or cytology test performed amongst direct referrals | *Histology or cytology test in direct referrals*, i.e. number of screen positives with ASC-US+ cytology where an examination was performed within 150 days from the screening test | Direct referrals |
| CIN2+ detection from direct referrals | Number of histological confirmed CIN2+ lesions | Histology or cytology test in direct referrals |
| Histology or cytology test performed in indirect referrals | *Histology or cytology test in indirect referrals*, i.e. number of follow-up smears with an ASC-US+ cytology result where an examination was performed within 150 days from follow-up smear. | Indirect referrals |
| CIN2+ rate from direct referrals | Number of histological confirmed CIN2+ lesions | Histology or cytology test in indirect referrals |

***Table A3:*** *Calculation of the indicators for participation, referral and detection in the old cytology-based screening programme, cohort 2015, and within the new hrHPV-based screening programme, 2017 cohort*.

| Indicator | Cohort | Numerator | Denominator |
| --- | --- | --- | --- |
| **Participation** |  |  |  |
| Participation rate  *Figure 2* | Cytology cohort | *Participants*, i.e. number of screening test results from clinician-based sampling, performed between 1 January 2015 and 31 March 2016. | *Eligible population*, i.e. number of women at screening ages in the Dutch population on 1 January 2015 and adjusted for the risk of having their cervix removed by hysterectomy. |
|  | hrHPV cohort | *Participants*, i.e. number of screening test results from clinician-based sampling or self-sampling, performed between 1 January 2017 and 30 June 2018. | *Eligible population*, i.e. number of women at screening ages in the Dutch population on 1 January 2017 and adjusted for the risk of having their cervix removed by hysterectomy. |
| **Referral** |  |  |  |
| Screen positivity  *Figure 3* | Cytology cohort | *Screen-positives*, i.e. number of screening tests with ASCUS+ cytology | Participants |
|  | hrHPV cohort | *Screen positives*, i.e. number of hrHPV positive screening tests | Participants |
| Referral rate from primary screening (direct referral)  *Figure 3, Figure 5, Table 1, Table 2* | Cytology cohort | *Direct referrals*, i.e. number of screen positive women with HSIL cytology result | Participants |
|  | hrHPV cohort | *Direct referrals,* i.e. number of hrHPV positive screening tests with ASCUS+ cytology result | Participants |
| Referral rate from follow-up smear (indirect referral)  *Figure 5, Table 1, Table 2* | Cytology cohort | *Indirect referrals*, Number of follow-up smears at first or second repeat cytology, with HSIL cytology result | Participants |
|  | hrHPV cohort | *Indirect referrals*, Number of follow-up smears with ASC-US+ cytology result | Participants |
| **Detection** |  |  |  |
| CIN2+  *Figure 5, Table 1, Table 2* | Cytology cohort | Number of CIN2+ lesions found in referred women (direct and indirect). | Participants |
|  | hrHPV cohort | Number of CIN2+ lesions found in referred women (direct and indirect). | Participants |
| All findings  *Table 1, Table 2* | Cytology cohort | Number of findings in referred women (direct and indirect). For cytology only group, women must have had an examination within 150 days of primary screening (direct referrals) or within 150 days of repeat cytology test (indirect referrals). | Participants |
|  | hrHPV cohort | Number of findings in referred women (direct and indirect). For cytology only group, women must have had an examination within 150 days of primary screening (direct referrals) or within 150 days of repeat cytology test (indirect referrals). | Participants |
